# Supplementary figures and images for: Exploring the Influence of the Selected Conjugated Fatty Acids Isomers and Cancerous Process on the Fatty Acids Profile of Spleen
Source: Cancers (Basel). 2024 Jan 23;16(3):479. doi: 10.3390/cancers16030479 (PMC10854539; doi:10.3390/cancers16030479)

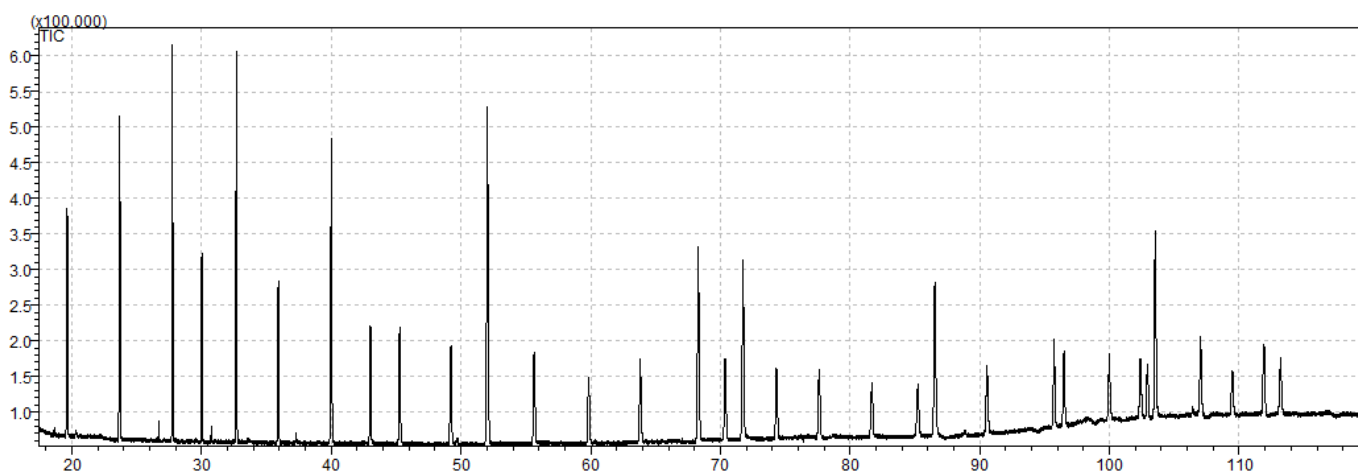

**Figure S1.** Chromatogram of FAME standards.

Supplement: Supplementary file 1 [file cancers-16-00479-s001.zip › cancers-2773419-supplementary.pdf]
